# Association between social vulnerability profiles, prenatal care use and pregnancy outcomes

**DOI:** 10.1186/s12884-023-05792-2

**Published:** 2023-06-22

**Authors:** Simon Crequit, Konstantinos Chatzistergiou, Gregory Bierry, Sakina Bouali, Adelaïde Dupre La Tour, Naima Sgihouar, Bruno Renevier

**Affiliations:** 1 Centre Hospitalier Intercommunal de Montreuil, 56 Boulevard de la Boissière, Montreuil, 93100 France; 2 GHT Grand Paris Nord Est, GHI Raincy Montfermeil, 10 rue du Général Leclerc, Montfermeil, 93370 France

**Keywords:** Social vulnerability, Social deprivation, Neonatal outcomes, Neonatal morbidity, SGA, Small for gestational age, Pregnancy outcomes, Premature birth

## Abstract

**Background:**

Evaluating social vulnerability is a challenging task. Indeed, former studies demonstrated an association between geographical social deprivation indicators, administrative indicators, and poor pregnancy outcomes.

**Objective:**

To evaluate the association between social vulnerability profiles, prenatal care use (PCU) and poor pregnancy outcomes (Preterm birth (PTB: <37 gestational weeks (GW)), small for gestational age (SGA), stillbirth, medical abortion, and late miscarriage).

**Methods:**

Retrospective single center study between January 2020 and December 2021. A total of 7643 women who delivered a singleton after 14 GW in a tertiary care maternity unit were included. Multiple component analysis (MCA) was used to assess the associations between the following social vulnerabilities: social isolation, poor or insecure housing conditions, not work-related household income, absence of standard health insurance, recent immigration, linguistic barrier, history of violence, severe dependency, psychologic vulnerability, addictions, and psychiatric disease. Hierarchical clustering on principal component (HCPC) from the MCA was used to classify patients into similar social vulnerability profiles. Associations between social vulnerability profiles and poor pregnancy outcomes were tested using multiple logistic regression or Poisson regression when appropriate.

**Results:**

The HCPC analysis revealed 5 different social vulnerability profiles. Profile 1 included the lowest rates of vulnerability and was used as a reference. After adjustment for maternal characteristics and medical factors, profiles 2 to 5 were independently associated with inadequate PCU (highest risk for profile 5, aOR = 3.14, 95%CI[2.33–4.18]), PTB (highest risk for profile 2, aOR = 4.64, 95%CI[3.80–5.66]) and SGA status (highest risk for profile 5, aOR = 1.60, 95%CI[1.20–2.10]). Profile 2 was the only profile associated with late miscarriage (adjusted incidence rate ratio (aIRR) = 7.39, 95%CI[4.17–13.19]). Profiles 2 and 4 were independently associated with stillbirth (highest association for profile 2 (aIRR = 10.9, 95%CI[6.11–19.99]) and medical abortion (highest association for profile 2 (aIRR = 12.65, 95%CI[5.96–28.49]).

**Conclusions:**

This study unveiled 5 clinically relevant social vulnerability profiles with different risk levels of inadequate PCU and poor pregnancy outcomes. A personalized patient management according to their profile could offer better pregnancy management and reduce adverse outcomes.

**Supplementary Information:**

The online version contains supplementary material available at 10.1186/s12884-023-05792-2.

## Background

Defining and measuring social vulnerability is a challenging task. Since the 80s, several studies have reported an association between social deprivation and poor pregnancy outcomes [1]. Indeed, socially deprived women present a higher risk of preterm birth (PTB) [2–7], an increased rate of small for gestational age (SGA) newborn [8] and stillbirth [8, 9]. In order to characterize social vulnerability, several studies aimed at defining deprivation scores to understand le link between social vulnerabilities and poor pregnancy outcomes. These scores were mainly based on administrative [10–12] or localization [3, 6, 8, 13, 14] indicators, patient level social vulnerabilities being rarely explored. Even if administrative scores have been validated and are easy to use [11, 12], they do not explain completely the gradients observed regarding adverse pregnancy outcomes and maternal origin or cultural factors [6, 9, 15]. Indeed, part of the observed differences could be explained by other stress factors such as interpersonal violence, recent immigration, psychological distress, linguistic barriers, and addictions. Recent studies reported that socially deprived women, based on administrative scores do not use properly prenatal care and that the differences in term of inadequate prenatal care use (PCU) could explain the increase in poor pregnancy outcomes [10, 16]. Even if reducing social vulnerabilities on administrative indicators is seducing and convenient, it remains unclear that other vulnerabilities apart from housing condition or health care insurance status also impact PCU and pregnancy outcomes. Therefore, in the present study, social vulnerability was defined by any stress factor that could negatively influence patient health literacy, pregnancy follow-up, and the detection or the management of pregnancy diseases. The aim of this study was to characterize social vulnerability profiles from a thorough collection of social vulnerabilities and assess the association between the different profiles PCU and poor pregnancy outcomes (PTB, SGA, stillbirth, medical abortion, and late miscarriage).

## Materials and methods

### Study population

The aim of the study was to provide an accurate estimation of social vulnerability profiles in the population covered by the maternity unit at study and their independent association with poor pregnancy outcomes. Therefore, we chose to include all the women with singleton pregnancy that could present the outcomes of interest. Twin pregnancies were excluded because they are known to be inherently associated with poor pregnancy outcomes, which would have biased the weight of the vulnerability profile in which they were mostly included.

Using birth records, a total of 7831 patients that delivered after 14 weeks of gestation between January 2020 and December 2021 in a single tertiary care maternity unit (CHI-Montreuil), were identified. After the exclusion of 188 twin pregnancies, 7643 patients with singleton pregnancies that delivered after 14 GW were included for analysis.

### Collected data

Data collection from patient’s informatized folder has already been described [17].

Social vulnerabilities were defined as follow: social isolation (absence of partner), Poor or insecure housing condition (no rented nor owned housing), no work-related household income (the woman’s household income came from public assistance, relatives, friends, or a charity), No permanent health care insurance (Couverture Maladie Universelle, CMU) or illegal status (Aide Médicale d’Etat, AME)), recent immigration (< 12 month), linguistic barrier, history of violence (interpersonal violence during pregnancy), severe dependency (with handicap or minor patient), addiction (Tobacco, alcohol, cannabis, cocaine derived drug and morphine derive drug use during pregnancy), psychological distress (pregnancy related anxiety, depressive symptoms or patient request for a psychologist follow-up) and psychiatric disease (major depressive disorder, bipolar disorder, post-traumatic stress disorder, Schizophrenia).

Inadequate prenatal care use (PCU) was defined as follow [10] : pregnancy follow up began after 12 weeks of gestation, or if it included less than 50% of the number of prenatal visits expected according to duration of pregnancy, or if the first-trimester ultrasound examination or both the second- and third- trimester examinations were missing.

SGA status was defined by a birthweight < to the 10th percentile according to the WHO fetal growth charts [18]. High medical risk level before pregnancy was defined as the presence of one or more of: history of cardiac disease, hypertension, diabetes, venous thrombosis, pulmonary embolism, Graves’ disease, asthma, homozygous sickle cell anemia, thrombocytopenia, coagulation disorder, a rare or systemic disease, nephropathy, HIV infection, psychiatric disease. High obstetrical risk level before pregnancy was defined by a history of one or more of the following: pre-eclampsia, fetal growth restriction, PTB, fetal death or neonatal death. Pregnancy complication was defined as the occurrence of one or more of the following complications: gestational diabetes, pre-eclampsia, fetal growth restriction, proteinuria, thrombopenia, threatened preterm labor, preterm premature rupture of membranes (PPROM), deep vein thrombosis and cholestasis of pregnancy.

### Statistical analysis

Maternal, pregnancy, labor, delivery, and neonatal characteristics were compared using Chi [2] or Fisher exact tests for categorical variables and Student’s or Wilcoxon rank sum tests for quantitative variables, as appropriate. All tests were two-sided with p-values < 0.05 defined as statistically significant. Multiple component analysis (MCA) was run on the 11 vulnerabilities aforementioned [19]. MCA allows to reduce the dimensions in interpreting the variability explained in a dataset by combining variables that are associated together into factorial axis (linear weighted combination of the variables included in the analysis) [11, 20]. The factorials axis being orthogonal it allows to avoid multicollinearity in regression analysis. The analysis of the MCA graphs did not detect any outlier within the population. The analysis of inertia suggested to restrict the analysis to the three first factorial axis that accounted for 47% of the total inertia. The three factorial axes were named according to their clinical relevance. To assess the association of a one-point increase in each index with poor pregnancy outcomes, multiple logistic regression models were run including the three indexes together as continuous variables.

In order to characterize social vulnerability profiles, Hierarchical Clustering on Principal Component (HCPC) was performed from the MCA analysis, using Ward’s method consolidated by k-means to merge similar patients into clusters [21]. The optimal number of clusters was determined by maximizing the ratio of between cluster inertia over the inertia increase of adding a cluster [19, 22]. This analysis resulted in five clusters that represented five distinct social vulnerability profiles.

The independent association between inadequate PCU, premature birth (< 37 GW), SGA status, and the social vulnerability profiles were tested using multiple logistic regression. Because stillbirth, late miscarriage and medical abortion were rare events within the profiles, the incidence rate ratio (IRR) were calculated using multiple Poisson regression. Adjustment was performed on maternal age, maternal origin, parity, BMI, high medical risk level before pregnancy and high obstetrical risk level before pregnancy. No multicollinearity was detected using variance inflation factor. Visual inspection of residual plots did not reveal any obvious deviations from homoscedasticity or normality. Factominer package [20] was used to perform MCA and HCPC analyses. R software (R Development Core Team (2008), version 4.2.0) was used for all analyses.

## Results

Among the 7643 patients included in the study (Table 1), 353 (4.6%) presented recent immigration, 2521 (33.0%) received not work-related household income, 2037 (26.7%) did not benefit from permanent health care insurance, 1128 (14.8%) had poor or insecure housing conditions, 546 (7.1%) had a history of interpersonal violence during pregnancy, 1279 (16.7%) were socially isolated, 1047 (13.7%) presented psychological distress, 492 (6.4%) had a linguistic barrier, 230 (3.0%) presented a severe dependency, 1315 (17.2%) presented addiction during pregnancy, and 236 (3.1%) had a psychiatric disease. The MCA results are summarized in Table 1. The variability regarding social vulnerabilities was well explained by the three first factorial axes. The first factorial axis accounted for 23.66% of the total inertia. It opposed patients presenting administrative vulnerabilities (recent immigration, not work-related household income, no permanent healthcare insurance, poor or insecure housing conditions), history of violence, social isolation and psychologic follow-up to the patient that didn’t present these vulnerabilities. It was therefore named the administrative social vulnerability index (AVI). The second axis presented an inertia of 12.91%. It contrasted patients that presented psychological distress, that had a psychiatric disease, that had a history of violence and addiction during pregnancy, received work related household income, were insured, that had proper housing conditions and didn’t present linguistic barrier. It was named the psychological vulnerability index (PVI). The third factorial axis presented an inertia of 10.38%. It contrasted patients that presented severe dependency, having poor or insecure housing conditions, being socially isolated, that didn’t present recent immigration, history of violence, psychiatric disease nor linguistic barrier. It was named the dependency vulnerability index (DVI). Description of the population according to their score on the three indexes are presented in additional Tables 1, 2 and 3. Associations between the vulnerability axes and poor pregnancy outcomes are presented in additional Table 4.

**Table 1:** Description of the three factorial axes of the multiple correspondence analysis (MCA)

Vulnerability	Category	N (%)	Weights of category^1^		
			Factorial axis 1	Factorial axis 2	Factorial axis 3
Recent immigration	Immigration < 12 month	353 (4.6)	**2.81**	-0.55	**-1.7**
	No recent immigration		-0.14	0.03	0.08
Household income	Not work-related household income	2521 (33.0)	**1**	**-0.46**	0.17
	Work-related household income		-0.49	0.23	-0.08
Social insurance	No permanent health care insurance	2037 (26.7)	**1.07**	**-0.61**	-0.11
	Being insured		-0.39	0.22	0.04
Housing condition	Poor or insecure housing condition	1128 (14.8)	**1.43**	-0.36	**0.88**
	Living in personal housing		-0.25	0.06	-0.15
History of violence	Interpersonal violence during pregnancy	546 (7.1)	**1.89**	**1.23**	**-1.39**
	No history of violence during pregnancy		-0.15	-0.09	0.11
Social isolation	Not living with a partner	1279 (16.7)	**1.13**	0.33	**0.8**
	Living with a partner		-0.23	-0.07	-0.16
Psychological distress^2^	Psychological distress during pregnancy	1047 (13.7)	**1.08**	**1.56**	-0.23
	No psychological distress during pregnancy		-0.17	-0.25	0.04
Linguistic barrier	Linguistic barrier during pregnancy	492 (6.4)	1.02	**-1.62**	**-0.93**
	No linguistic barrier during pregnancy		-0.07	0.11	0.06
Severe dependency	Minor or with handicap patient	230 (3.0)	1.43	0.86	**3.56**
	Major patient without handicap		-0.04	-0.03	-0.11
Addiction^3^	Addiction during pregnancy	1315 (17.2)	0.18	**0.87**	0.49
	No history of addiction during pregnancy		-0.04	-0.18	-0.1
Psychiatric disease^4^	Psychiatric disease during pregnancy	236 (3.1)	1.73	**2.81**	**-1.39**
	No psychiatric disease during pregnancy		-0.06	-0.09	0.04

^1^The weights correspond to the category coordinates on the axes. Categories contributing to the axis are in bold

^2^Defined by pregnancy related anxiety, depressive symptoms or patient request for a psychologist follow-up

^3^Tobacco, alcohol, cannabis, cocaine derived drug or morphine derive drug use during pregnancy

^4^Major depressive disorder, bipolar disorder, post-traumatic stress disorder, Schizophrenia

**Table 2:** Maternal characteristics and pregnancy outcomes according to social vulnerability profiles

	Profile 1	Profile 2	Profile 3	Profile 4	Profile 5	p
	N = 4651	N = 773	N = 1597	N = 329	N = 293	
**Maternal Characteristics**						
Maternal age (median [IQR])	31.49 [28.03, 35.10]	32.27 [28.05, 36.22]	29.70 [25.47, 34.33]	26.98 [20.99, 32.58]	29.76 [25.35, 34.62]	< 0.001
BMI (median [IQR])	24.98 [22.27, 28.70]	25.16 [22.59, 29.34]	25.24 [22.58, 28.65]	24.44 [22.22, 28.16]	25.08 [23.63, 28.71]	0.257
Parity (median [IQR])	1.00 [0.00, 2.00]	1.00 [0.00, 2.00]	1.00 [0.00, 2.00]	1.00 [0.00, 2.00]	1.00 [0.00, 2.00]	< 0.001
Maternal origin n (%)						< 0.001
Sub-Saharan Africa	1617 ( 34.8)	255 ( 33.0)	626 ( 39.2)	150 ( 45.6)	167 ( 57.0)	
Asia	572 ( 12.3)	111 ( 14.4)	187 ( 11.7)	39 ( 11.9)	23 ( 7.8)	
Caucasian	2462 ( 52.9)	407 ( 52.7)	784 ( 49.1)	140 ( 42.6)	103 ( 35.2)	
High medical risk level before pregnancy^1^ n (%)	387 ( 8.3)	196 ( 25.4)	139 ( 8.7)	41 ( 12.5)	84 ( 28.7)	< 0.001
High obstetrical risk level before pregnancy^2^ n (%)	1180 ( 25.4)	269 ( 34.8)	412 ( 25.8)	79 ( 24.0)	74 ( 25.3)	< 0.001
Inadequate PCU^3^ n (%)	387 ( 8.3)	85 ( 11.0)	300 ( 18.8)	76 ( 23.1)	75 ( 25.6)	< 0.001
Unwanted pregnancy n (%)	14 ( 0.3)	15 ( 2.1)	5 ( 0.3)	13 ( 4.0)	9 ( 3.1)	< 0.001
**Social vulnerabilities**						
Immigration < 12 month n (%)	0 ( 0.0)	0 ( 0.0)	145 ( 9.1)	6 ( 1.8)	202 ( 68.9)	< 0.001
Not work-related household income n (%)	452 ( 9.7)	148 ( 19.1)	1409 ( 88.2)	231 ( 70.2)	281 ( 95.9)	< 0.001
No permanent health care insurance n (%)	343 ( 7.4)	84 ( 10.9)	1183 ( 74.1)	154 ( 46.8)	273 ( 93.2)	< 0.001
Poor or insecure housing condition n (%)	57 ( 1.2)	51 ( 6.6)	703 ( 44.0)	187 ( 56.8)	130 ( 44.4)	< 0.001
Not living with a partner n (%)	308 ( 6.6)	137 ( 17.7)	460 ( 28.8)	205 ( 62.3)	169 ( 57.7)	< 0.001
Psychological distress^4^ n (%)	7 ( 0.2)	606 ( 78.4)	60 ( 3.8)	154 ( 46.8)	220 ( 75.1)	< 0.001
Psychiatric disease^5^ n (%)	0 ( 0.0)	138 ( 17.9)	0 ( 0.0)	11 ( 3.3)	87 ( 29.7)	< 0.001
Addiction^6^ n (%)	712 ( 15.3)	233 ( 30.1)	173 ( 10.8)	144 ( 43.8)	53 ( 18.1)	< 0.001
Severe dependency n (%)	0 ( 0.0)	3 ( 0.4)	4 ( 0.3)	219 ( 66.6)	4 ( 1.4)	< 0.001
Linguistic barrier n (%)	94 ( 2.0)	4 ( 0.5)	328 ( 20.5)	9 ( 2.7)	57 ( 19.5)	< 0.001
History of violence n (%)	0 ( 0.0)	230 ( 29.8)	55 ( 3.4)	26 ( 7.9)	235 ( 80.2)	< 0.001
**Pregnancy outcomes**						
Pregnancy complications^7^ n (%)	2686 ( 57.8)	548 ( 70.9)	1055 ( 66.1)	207 ( 62.9)	197 ( 67.2)	< 0.001
Premature birth (< 37 weeks) n (%)	317 ( 6.8)	216 ( 27.9)	146 ( 9.1)	48 ( 14.6)	33 ( 11.3)	< 0.001
SGA^8^ n (%)	838 ( 18.0)	192 ( 24.8)	322 ( 20.2)	81 ( 24.6)	77 ( 26.3)	< 0.001
Pregnancy outcome n (%)						
Neonatal death	0 ( 0.0)	0 ( 0.0)	1 ( 0.1)	0 ( 0.0)	1 ( 0.3)	NA
Late miscarriage	23 ( 0.5)	26 ( 3.4)	7 ( 0.4)	3 ( 0.9)	0 ( 0.0)	< 0.001
Medical abortion	10 ( 0.2)	20 ( 2.6)	6 ( 0.4)	3 ( 0.9)	2 ( 0.7)	< 0.001
Stillbirth	18 ( 0.4)	33 ( 4.3)	12 ( 0.8)	5 ( 1.5)	1 ( 0.3)	< 0.001

^1^defined as the presence of one or more of: history of cardiac disease, hypertension, diabetes, venous thrombosis, pulmonary embolism, Graves’ disease, asthma, homozygous sickle cell anemia, thrombocytopenia, coagulation disorder, a rare or systemic disease, nephropathy, HIV infection

^2^defined by a history of one or more of the following: pre-eclampsia, fetal growth restriction, preterm delivery, fetal or neonatal death

^3^pregnancy follow-up began after 12 weeks of gestation, or if it included less than 50% of the number of prenatal visits expected according to duration of pregnancy, or if the first-trimester ultrasound examination or both the second- and third- trimester examinations were missing

^4^Defined by pregnancy related anxiety, depressive symptoms, or patient request for a psychologist follow-up

^5^Major depressive disorder, bipolar disorder, post-traumatic stress disorder, Schizophrenia

^6^Tobacco, alcohol, cannabis, cocaine derived drug and morphine derive drug use during pregnancy ^7^defined as the occurrence of one or more of the following complications: gestational diabetes, pre-eclampsia, fetal growth restriction, proteinuria, thrombopenia, threatened preterm labor, premature rupture of membranes (PROM), deep vein thrombosis and cholestasis of pregnancy

^8^Small for gestational was defined by a birthweight < to the 10th percentile according to the WHO fetal growth charts

**Table 3:** Association between social vulnerability profiles and poor pregnancy outcomes

	**Inadequate prenatal care use**	
	**OR**	**aOR**
Profile 1	ref	ref
Profile 2	1.36 [1.06–1.74]*	1.37 [1.06–1.76]*
Profile 3	2.55 [2.16–3.00]***	2.36 [2.00–2.78]***
Profile 4	3.31 [2.50–4.35]***	2.94 [2.20–3.89]***
Profile 5	3.79 [2.84–5.01]***	3.14 [2.33–4.18]***
	**Premature birth (< 37 weeks)**	
	**OR**	**aOR**
Profile 1	ref	ref
Profile 2	5.30 [4.36–6.43]***	4.64 [3.80–5.66]***
Profile 3	1.38 [1.12–1.69]**	1.44 [1.17–1.77]**
Profile 4	2.34 [1.67–3.21]***	2.54 [1.80–3.52]***
Profile 5	1.74 [1.17–2.50]**	1.67 [1.12–2.44]**
	**Small for gestational age** ^[[1]]^	
	**OR**	**aOR**
Profile 1	ref	ref
Profile 2	1.50 [1.25–1.80]***	1.47 [1.22–1.76]***
Profile 3	1.15 [0.99–1.33]	1.17 [1.01–1.35]*
Profile 4	1.49 [1.14–1.92]**	1.44 [1.10–1.88]**
Profile 5	1.62 [1.23–2.12]***	1.60 [1.20–2.10]**
	**Stillbirth**	
	**IRR**	**aIRR**
Profile 1	ref	ref
Profile 2	11.0 [6.29–19.99]***	10.9 [6.11–19.99]***
Profile 3	1.94 [0.91–3.99]	1.88 [0.88–3.89]
Profile 4	3.93 [1.30–9.84]**	3.98 [1.30–10.12]**
Profile 5	0.88 [0.05–4.27]	0.82 [0.05–4.03]
	**Late miscarriage**	
	**IRR**	**aIRR**
Profile 1	ref	ref
Profile 2	6.80 [3.88–12.00]***	7.39 [4.17–13.19]***
Profile 3	0.89 [0.35–1.96]	0.90 [0.36–2.01]
Profile 4	1.84 [0.44–5.30]	1.91 [0.45–5.62]
Profile 5	0.00 [0.00–0.00]	0.00 [0.00–0.00]
	**Medical abortion**	
	**IRR**	**aIRR**
Profile 1	ref	ref
Profile 2	12.03 [5.76–26.81]***	12.65 [5.96–28.49]***
Profile 3	1.75 [0.59–4.71]	1.99 [0.68–5.38]
Profile 4	4.24 [1.05–13.86]*	5.78 [1.28–19.19]**
Profile 5	3.17 [0.49–12.04]	3.89 [0.59–15.10]

^1^Small for gestational was defined by a birthweight < to the 10th percentile according to the WHO fetal growth charts

OR: Odd ratio

aOR: Adjusted OR, adjustment on maternal age, parity, BMI, high medical risk level before pregnancy and high obstetrical risk level before pregnancy

IRR: Incidence rate ratio

aIRR : Adjusted IRR,, adjustment on maternal age, maternal origin, parity, BMI, high medical risk level before pregnancy and high obstetrical risk level before pregnancy

*p < 0.05, **p < 0.01,***p < 0.001

The HCPC analysis revealed 5 different social vulnerability profiles (Table 2). Profile 1 included mainly Caucasian women with low rates of social vulnerabilities. Profile 2 was also characterized by mainly Caucasian women with high rates of psychological distress, interpersonal violence, psychiatric disease, and addictions whereas administrative vulnerability indicators rates were low. Profile 3 included mainly Caucasian women with high administrative vulnerability indicators that presented a linguistic barrier and low rates of psychologic vulnerability indicators. Profile 4 was characterized primarily by a Sub-saharian population with high prevalence of administrative, dependency and psychological vulnerability indicators. Finally, profile 5 included mainly a Sub-Saharan population with recent migration status and presented the highest rates of administrative vulnerability indicators and interpersonal violence while the rate of psychologic vulnerability indicators within the group was also high. Compared to women included in profile 1, profile’s 2 patients were older, whereas patients included in profile 3 to 5 were younger. Regarding medical factors, high medical and obstetrical risk level before pregnancy were more prevalent in profile 2 and 5. Profile 5 also presented the highest rate of pregnancy complications. Inadequate PCU rates increased from profile 1 to profile 5. Profiles 2 to 5 presented higher rates of PTB and small for gestational age neonates compared to profile 1. Late miscarriage and stillbirth were more frequent in profile 2 and 4. Profile 2 to 5 presented higher rates of medical abortion compared to profile 1.

After adjustment for maternal characteristics and medical factors, each profile was independently associated with inadequate PCU (Profile 1 as a reference, Table 3), with highest risk for profile 5 (aOR = 3.14, 95%CI[2.33–4.18]). Profiles 2 to 5 were also independently associated with PTB (highest association for profile 2 (aOR = 4.64, 95%CI[3.80–5.66])). The latter, were also independently associated with SGA status and the strongest association was for profile 5 (aOR = 1.60, 95%CI[1.20–2.10]). Profile 2 was the only profile associated with late miscarriage (adjusted incidence rate ratio (aIRR) = 7.39, 95%CI[4.17–13.19]). Profiles 2 and 4 were independently associated with stillbirth (highest association for profile 2 (aIRR = 10.9, 95%CI[6.11–19.99])) and medical abortion (strongest association for profile 2 (aIRR = 12.65, 95%CI[5.96–28.49])).

## Discussion

This work gives new insight on social vulnerability profiles and their association to prenatal care use and pregnancy outcomes. The three vulnerability axes defined by MCA analysis allow a better understanding of the associations between social vulnerabilities and contrasted relevant associations. The HCPC analysis identified 5 distinct clinically relevant profiles with different risk level of adverse pregnancy outcomes.

To our knowledge, this study is the first to gather such a large sample of patient-based data. Therefore, authentic associations between social vulnerabilities were discovered and produced a degree of precision regarding social vulnerability profiles that was never reached before. Another striking issue is the clinical relevance of the produced profiles for the clinician in the maternity unit at study. Indeed, profile 5 is highly representative of refugee women whereas profile 3 is more representative of stable migrants or native women low socio-economic status (SES). Profile 4 is representative of women with handicap and profile 2 represents women with high SES that presents high degrees of psychological distress and addictions.

Our finding regarding the association between profile 5 (refugee women) and PTB is consistent with a systematic review that demonstrated that PTB was the most frequent adverse pregnancy outcome in this population [23]. A recent study carried out in both Belgium and Canada studied the association between PTB, low SES and immigration status [24]. The authors demonstrated that low SES was associated with PTB, but there were differences between the two countries at study regarding this association and immigration status. The result is consistent with our findings as profile 3 and 5 were associated with PTB. Yet, this difference regarding immigration status was attributed to health inequalities between these countries. We show that profile 5 patients (refugee women) presented additional psychological vulnerabilities and violence compared to profile 3 patients (stable migrants or native women with administrative vulnerability). Part of the differences they found might be explained by distinct proportions of these two vulnerability profiles that were not explored. Similarly, two recent studies from the US demonstrated strong associations between social vulnerability indices and PTB [25, 26]. Both indices were based on geographical indicators of social vulnerability that blended several dimensions of social vulnerability together. Our approach gives further insight to these results as the associations we found comes from authentic patient level. Indeed, we show that profile 2 patients (high SES and psychological vulnerabilities) present a higher risk of PTB compared to profile 5 patients (refugee women) whereas these latter present a higher risk of SGA newborns.

The fact that these relevant profiles are at risk of different adverse pregnancy outcomes emphasizes that social vulnerability cannot be reduced to administrative factors only and that prevention measures should be adapted to the patient’s profile.

### Clinical implications

This study confirms the strong association between inadequate PCU and administrative vulnerability indicators that was found in previous works [10, 16]. Profiles 2 to 5 presented increasing degrees of administrative vulnerability that was parallel to the increase of inadequate PCU. Yet, our results challenge the hypothesis that PCU improvement would be the only answer to improve pregnancy outcomes. Indeed, profiles 2 and 4 presented lower degrees of inadequate PCU compared to profile 5. Yet, they presented higher risks for adverse outcomes. A recent analysis has shown that only 64% of French maternity unit offers prenatal interviews to detect maternal social vulnerability with limited access for deprived women [27]. This point accentuates that efforts must be made in the detection of patients’ vulnerability profiles to implement measures adapted to the actual risks. For instance, caseload midwifery has proven to have a positive effect on preterm birth for patients that presented the lowest quintiles of SES in London. Finally, a novel approach of personalized follow-up according to the patients social vulnerabilities had a similar effect [17].

### Strength and limitations

The main strength of this work comes from its large sample size and a reliable and complete data collection on maternal social vulnerabilities from the patient’s computerized medical folder whose content was checked in staff after each delivery. To our knowledge, this study was the first to produce social vulnerability profiles from such a large panel of different patient level social vulnerabilities. Thorough access to medical data allowed to adjust on medical and demographic confusion factors in multiple regression modelling.

However, this work presents several limitations. The experimental design of the study lead to the creation of composite variables such as psychological distress, addictions, or psychiatric diseases. Indeed, adding each of the item composing them in the MCA analysis separately would create noise: very small sub-groups of multicollinear variables that would lead to uncertainty in both the HCPC clustering (creation of non-relevant clusters) and multiple logistic regression (instability in coefficients estimation). Yet, using all the vulnerabilities items separately in a multiple logistic regression model would be biased and misleading.

Moreover, the sample selection with the inclusion of all singleton deliveries might reduce the generalization of the results, even if thorough adjustments on confusion factors were performed in the multiple regression models. The maternity unit at study is located in an area with high rate of socially deprived women. Moreover, patients were included in a tertiary care maternity unit and presented a large proportion of patients with high obstetrical and medical risk level before pregnancy along with a high rate of pregnancy complications. Finally, stillbirth, late miscarriage and medical abortion were rare events and even the use of Poisson regression produced large confidence intervals. Further studies with larger sample should improve this flaw.

## Conclusions

Altogether, these results suggest that social vulnerability cannot be reduced to administrative indicators only. Indeed, profiles with high rates of psychological vulnerability indicators were independently correlated to higher risk for poor pregnancy outcomes. Therefore, prevention measures should take into consideration a more spherical approach of the vulnerability profile and also target specific non-administrative stress factors.

## Electronic supplementary material

Below is the link to the electronic supplementary material.

Supplementary Material 1

## Data Availability

The datasets used and/or analyzed during the current study are available from the corresponding author on reasonable request.

## Abbreviations

AVI: Administrative vulnerability index
PVI: Psychologic vulnerability index
DVI: Dependency vulnerability index
SGA: Small for gestational age
PCU: Prenatal care use
BMI: Body mass index
OR: Odd ratio
aOR: Adjusted Odd ratio
IRR: Incidence rate ratio
aIRR: Adjusted incidence rate ratio
