# Supplementary material for: Association between social vulnerability profiles, prenatal care use and pregnancy outcomes
Source: BMC Pregnancy Childbirth. 2023 Jun 22;23:465. doi: 10.1186/s12884-023-05792-2 (PMC10288786; doi:10.1186/s12884-023-05792-2)
Supplement: Supplementary file 1 — Supplementary Material 1 [file 12884_2023_5792_MOESM1_ESM.docx]

Additional table 1: Maternal characteristics and pregnancy outcomes according to administrative vulnerability index scores.

| Administrative vulnerability index score | Low^1^ | Medium^2^ | High^3^ | p |
| --- | --- | --- | --- | --- |
| **Maternal Characteristics** | N=5809 | N=922 | N=912 |  |
| Maternal age (median [IQR]) | 31.43 [27.78, 35.20] | 29.90 [25.29, 34.34] | 29.15 [24.51, 34.12] | <0.001 |
| BMI (median [IQR]) | 25.00 [22.41, 28.73] | 25.24 [22.16, 29.29] | 24.46 [22.49, 28.18] | 0.296 |
| Parity (median [IQR]) | 1.00 [0.00, 2.00] | 1.00 [0.00, 2.00] | 1.00 [0.00, 2.00] | 0.623 |
| Maternal origin n (%) |  |  |  | <0.001 |
| Sub-Saharan Africa | 2017 (34.7) | 365 (39.6) | 433 (47.5) |  |
| Asia | 719 (12.4) | 114 (12.4) | 99 (10.9) |  |
| Caucasian | 3073 (52.9) | 443 (48.0) | 380 (41.7) |  |
| High medical risk level before pregnancy^4^ n (%) | 545 ( 9.4) | 124 (13.4) | 178 (19.5) | <0.001 |
| High obstetrical risk level before pregnancy^5^ n (%) | 1538 (26.5) | 251 (27.2) | 225 (24.7) | 0.420 |
| Inadequate PCU^6^ n (%) | 528 ( 9.1) | 145 (15.7) | 250 (27.4) | <0.001 |
| Unwanted pregnancy n (%) | 23 ( 0.4) | 10 ( 1.1) | 23 ( 2.6) | <0.001 |
| **Social vulnerabilities** |  |  |  |  |
| Immigration < 12 month n (%) | 0 ( 0.0) | 0 ( 0.0) | 353 (38.7) | <0.001 |
| Not work-related household income n (%) | 940 (16.2) | 737 (79.9) | 844 (92.5) | <0.001 |
| No permanent health care insurance n (%) | 848 (14.6) | 438 (47.5) | 751 (82.3) | <0.001 |
| Poor or insecure housing condition n (%) | 124 ( 2.1) | 441 (47.8) | 563 (61.7) | <0.001 |
| Not living with a partner n (%) | 481 ( 8.3) | 292 (31.7) | 506 (55.5) | <0.001 |
| Psychological distress^7^ n (%) | 441 ( 7.6) | 190 (20.6) | 416 (45.6) | <0.001 |
| Psychiatric disease^8^ n (%) | 59 ( 1.0) | 48 ( 5.2) | 129 (14.1) | <0.001 |
| Addiction^9^ n (%) | 854 (14.7) | 251 (27.2) | 210 (23.0) | <0.001 |
| Severe dependency^10^ n (%) | 47 ( 0.8) | 50 ( 5.4) | 133 (14.6) | <0.001 |
| Linguistic barrier n (%) | 199 ( 3.4) | 118 (12.8) | 175 (19.2) | <0.001 |
| History of violence n (%) | 56 ( 1.0) | 104 (11.3) | 386 (42.3) | <0.001 |
| **Pregnancy outcomes** |  |  |  |  |
| Pregnancy complications^11^ n (%) | 3449 (59.4) | 629 (68.2) | 615 (67.4) | <0.001 |
| Premature birth (<37 weeks) n (%) | 523 ( 9.0) | 115 (12.5) | 122 (13.4) | <0.001 |
| SGA^12^ n (%) | 1076 (18.5) | 214 (23.2) | 220 (24.1) | <0.001 |
| Pregnancy outcome n (%) |  |  |  | 0.131 |
| Neonatal death | 0 ( 0.0) | 1 ( 0.1) | 1 ( 0.1) |  |
| Late miscarriage | 47 ( 0.8) | 7 ( 0.8) | 5 ( 0.5) |  |
| Medical abortion | 27 ( 0.5) | 6 ( 0.7) | 8 ( 0.9) |  |
| Stillbirth | 47 ( 0.8) | 10 ( 1.1) | 12 ( 1.3) |  |

^1^Defined by a negative score or a score ≤ to the 33^rd^ percentile of positive scores.

^2^Defined by a score > to the 33^rd^ and ≤ to the 66^th^ percentile of positive scores.

^3^Defined by a score > to the 66^th^ percentile of positive scores.

^4^defined as the presence of one or more of: history of cardiac disease, hypertension, diabetes, venous thrombosis, pulmonary embolism, Graves’ disease, asthma, homozygous sickle cell anemia, thrombocytopenia, coagulation disorder, a rare or systemic disease, nephropathy, HIV infection.

^5^defined by a history of one or more of the following: pre-eclampsia, fetal growth restriction, preterm delivery, fetal or neonatal death.

^6^pregnancy follow-up began after 12 weeks of gestation, or if it included less than 50% of the number of prenatal visits expected according to duration of pregnancy, or if the first-trimester ultrasound examination or both the second- and third- trimester examinations were missing.

^7^Defined by pregnancy related anxiety, depressive symptoms, or patient request for a psychologist follow-up.

^8^Major depressive disorder, bipolar disorder, post-traumatic stress disorder, Schizophrenia.

^9^Tobacco, alcohol, cannabis, cocaine derived drug and morphine derived drug use during pregnancy.

^10^Minor or with handicap patient.

^11^defined as the occurrence of one or more of the following complications: gestational diabetes, pre-eclampsia, fetal growth restriction, proteinuria, thrombopenia, threatened preterm labor, premature rupture of membranes (PROM), deep vein thrombosis and cholestasis of pregnancy.

^12^Small for gestational was defined by a birthweight < to the 10^th^ percentile according to the WHO fetal growth charts.

Additional table 2: Maternal characteristics and pregnancy outcomes according to Pyschological vulnerability index scores.

| Psychological vulnerability index score | Low^1^ | Medium^2^ | High^3^ | p |
| --- | --- | --- | --- | --- |
| **Maternal Characteristics** | 6012 | 1028 | 603 |  |
| Maternal age (median [IQR]) | 30.94 [27.10, 34.88] | 31.52 [27.62, 35.37] | 31.36 [26.61, 35.59] | 0.044 |
| BMI (median [IQR]) | 25.00 [22.49, 28.71] | 24.84 [21.88, 28.65] | 24.91 [22.27, 29.34] | 0.156 |
| Parity (median [IQR]) | 1.00 [0.00, 2.00] | 1.00 [0.00, 2.00] | 1.00 [0.00, 2.00] | <0.001 |
| Maternal origin n (%) |  |  |  | <0.001 |
| Sub-Saharan Africa | 2266 (37.7) | 305 (29.7) | 244 (40.5) |  |
| Asia | 697 (11.6) | 159 (15.5) | 76 (12.6) |  |
| Caucasian | 3049 (50.7) | 564 (54.9) | 283 (46.9) |  |
| High medical risk level before pregnancy^4^ n (%) | 504 ( 8.4) | 131 (12.7) | 212 (35.2) | <0.001 |
| High obstetrical risk level before pregnancy^5^ n (%) | 1519 (25.3) | 295 (28.7) | 200 (33.2) | <0.001 |
| Inadequate PCU^6^ n (%) | 742 (12.3) | 92 ( 8.9) | 89 (14.8) | 0.001 |
| Unwanted pregnancy n (%) | 27 ( 0.5) | 13 ( 1.3) | 16 ( 2.7) | <0.001 |
| **Social vulnerabilities** |  |  |  |  |
| Immigration < 12 month n (%) | 283 ( 4.7) | 24 ( 2.3) | 46 ( 7.6) | <0.001 |
| Not work-related household income n (%) | 2143 (35.6) | 147 (14.3) | 231 (38.3) | <0.001 |
| No permanent health care insurance n (%) | 1794 (29.8) | 91 ( 8.9) | 152 (25.2) | <0.001 |
| Poor or insecure housing condition n (%) | 921 (15.3) | 64 ( 6.2) | 143 (23.7) | <0.001 |
| Not living with a partner n (%) | 874 (14.5) | 163 (15.9) | 242 (40.1) | <0.001 |
| Psychological distress^7^ n (%) | 169 ( 2.8) | 362 (35.2) | 516 (85.6) | <0.001 |
| Psychiatric disease^8^ n (%) | 10 ( 0.2) | 7 ( 0.7) | 219 (36.3) | <0.001 |
| Addiction^9^ n (%) | 379 ( 6.3) | 628 (61.1) | 308 (51.1) | <0.001 |
| Severe dependency^10^ n (%) | 97 ( 1.6) | 36 ( 3.5) | 97 (16.1) | <0.001 |
| Linguistic barrier n (%) | 474 ( 7.9) | 5 ( 0.5) | 13 ( 2.2) | <0.001 |
| History of violence n (%) | 198 ( 3.3) | 103 (10.0) | 245 (40.6) | <0.001 |
| **Pregnancy outcomes** |  |  |  |  |
| Pregnancy complications^11^ n (%) | 3625 (60.3) | 640 (62.3) | 428 (71.0) | <0.001 |
| Premature birth (<37 weeks) n (%) | 458 ( 7.6) | 177 (17.2) | 125 (20.7) | <0.001 |
| SGA^12^ n (%) | 1119 (18.6) | 233 (22.7) | 158 (26.2) | <0.001 |
| Pregnancy outcome n (%) |  |  |  | <0.001 |
| Neonatal death | 1 ( 0.0) | 0 ( 0.0) | 1 ( 0.2) |  |
| Late miscarriage | 30 ( 0.5) | 24 ( 2.3) | 5 ( 0.8) |  |
| Medical abortion | 18 ( 0.3) | 9 ( 0.9) | 14 ( 2.3) |  |
| Stillbirth | 34 ( 0.6) | 19 ( 1.8) | 16 ( 2.7) |  |

^1^Defined by a negative score or a score ≤ to the 33^rd^ percentile of positive scores.

^2^Defined by a score > to the 33^rd^ and ≤ to the 66^th^ percentile of positive scores.

^3^Defined by a score > to the 66^th^ percentile of positive scores.

^4^defined as the presence of one or more of: history of cardiac disease, hypertension, diabetes, venous thrombosis, pulmonary embolism, Graves’ disease, asthma, homozygous sickle cell anemia, thrombocytopenia, coagulation disorder, a rare or systemic disease, nephropathy, HIV infection.

^5^defined by a history of one or more of the following: pre-eclampsia, fetal growth restriction, preterm delivery, fetal or neonatal death.

^6^pregnancy follow-up began after 12 weeks of gestation, or if it included less than 50% of the number of prenatal visits expected according to duration of pregnancy, or if the first-trimester ultrasound examination or both the second- and third- trimester examinations were missing.

^7^Defined by pregnancy related anxiety, depressive symptoms, or patient request for a psychologist follow-up.

^8^Major depressive disorder, bipolar disorder, post-traumatic stress disorder, Schizophrenia.

^9^Tobacco, alcohol, cannabis, cocaine derived drug and morphine derived drug use during pregnancy.

^10^Minor or with handicap patient.

^11^defined as the occurrence of one or more of the following complications: gestational diabetes, pre-eclampsia, fetal growth restriction, proteinuria, thrombopenia, threatened preterm labor, premature rupture of membranes (PROM), deep vein thrombosis and cholestasis of pregnancy.

^12^Small for gestational was defined by a birthweight < to the 10^th^ percentile according to the WHO fetal growth charts.

Additional table 3: Maternal characteristics and pregnancy outcomes according to Dependency vulnerability index scores.

| Dependency vulnerability index score | Low^1^ | Medium^2^ | High^3^ | p |
| --- | --- | --- | --- | --- |
| **Maternal Characteristics** | 5496 | 1269 | 878 |  |
| Maternal age (median [IQR]) | 31.37 [27.72, 35.14] | 30.97 [26.86, 35.10] | 28.31 [24.08, 33.44] | <0.001 |
| BMI (median [IQR]) | 25.00 [22.49, 28.72] | 25.14 [22.05, 28.52] | 24.90 [22.21, 28.96] | 0.579 |
| Parity (median [IQR]) | 1.00 [0.00, 2.00] | 1.00 [0.00, 2.00] | 1.00 [0.00, 2.00] | <0.001 |
| Maternal origin n (%) |  |  |  | <0.001 |
| Sub-Saharan Africa | 1965 (35.8) | 466 (36.7) | 384 (43.7) |  |
| Asia | 652 (11.9) | 179 (14.1) | 101 (11.5) |  |
| Caucasian | 2879 (52.4) | 624 (49.2) | 393 (44.8) |  |
| High medical risk level before pregnancy^4^ n (%) | 617 (11.2) | 131 (10.3) | 99 (11.3) | 0.640 |
| High obstetrical risk level before pregnancy^5^ n (%) | 1447 (26.3) | 359 (28.3) | 208 (23.7) | 0.059 |
| Inadequate PCU^6^ n (%) | 620 (11.3) | 137 (10.8) | 166 (18.9) | <0.001 |
| Unwanted pregnancy n (%) | 32 ( 0.6) | 5 ( 0.4) | 19 ( 2.2) | <0.001 |
| **Social vulnerabilities** |  |  |  |  |
| Immigration < 12 month n (%) | 341 ( 6.2) | 3 ( 0.2) | 9 ( 1.0) | <0.001 |
| Not work-related household income n (%) | 1537 (28.0) | 360 (28.4) | 624 (71.1) | <0.001 |
| No permanent health care insurance n (%) | 1415 (25.7) | 346 (27.3) | 276 (31.4) | 0.002 |
| Poor or insecure housing condition n (%) | 307 ( 5.6) | 253 (19.9) | 568 (64.7) | <0.001 |
| Not living with a partner n (%) | 285 ( 5.2) | 444 (35.0) | 550 (62.6) | <0.001 |
| Psychological distress^7^ n (%) | 775 (14.1) | 94 ( 7.4) | 178 (20.3) | <0.001 |
| Psychiatric disease^8^ n (%) | 211 ( 3.8) | 9 ( 0.7) | 16 ( 1.8) | <0.001 |
| Addiction^9^ n (%) | 322 ( 5.9) | 659 (51.9) | 334 (38.0) | <0.001 |
| Severe dependency^10^ n (%) | 0 ( 0.0) | 2 ( 0.2) | 228 (26.0) | <0.001 |
| Linguistic barrier n (%) | 439 ( 8.0) | 27 ( 2.1) | 26 ( 3.0) | <0.001 |
| History of violence n (%) | 495 ( 9.0) | 30 ( 2.4) | 21 ( 2.4) | <0.001 |
| **Pregnancy outcomes** |  |  |  |  |
| Pregnancy complications^11^ n (%) | 3329 (60.6) | 786 (61.9) | 578 (65.8) | 0.011 |
| Premature birth (<37 weeks) n (%) | 508 ( 9.2) | 135 (10.6) | 117 (13.3) | 0.001 |
| SGA^12^ n (%) | 1045 (19.0) | 268 (21.1) | 197 (22.4) | 0.025 |
| Pregnancy outcome n (%) |  |  |  | 0.330 |
| Neonatal death | 1 ( 0.0) | 1 ( 0.1) | 0 ( 0.0) |  |
| Late miscarriage | 49 ( 0.9) | 5 ( 0.4) | 5 ( 0.6) |  |
| Medical abortion | 26 ( 0.5) | 10 ( 0.8) | 5 ( 0.6) |  |
| Stillbirth | 50 ( 0.9) | 14 ( 1.1) | 5 ( 0.6) |  |

^1^Defined by a negative score or a score ≤ to the 33^rd^ percentile of positive scores.

^2^Defined by a score > to the 33^rd^ and ≤ to the 66^th^ percentile of positive scores.

^3^Defined by a score > to the 66^th^ percentile of positive scores.

^4^defined as the presence of one or more of: history of cardiac disease, hypertension, diabetes, venous thrombosis, pulmonary embolism, Graves’ disease, asthma, homozygous sickle cell anemia, thrombocytopenia, coagulation disorder, a rare or systemic disease, nephropathy, HIV infection.

^5^defined by a history of one or more of the following: pre-eclampsia, fetal growth restriction, preterm delivery, fetal or neonatal death.

^6^pregnancy follow-up began after 12 weeks of gestation, or if it included less than 50% of the number of prenatal visits expected according to duration of pregnancy, or if the first-trimester ultrasound examination or both the second- and third- trimester examinations were missing.

^7^Defined by pregnancy related anxiety, depressive symptoms, or patient request for a psychologist follow-up.

^8^Major depressive disorder, bipolar disorder, post-traumatic stress disorder, Schizophrenia.

^9^Tobacco, alcohol, cannabis, cocaine derived drug and morphine derived drug use during pregnancy.

^10^Minor or with handicap patient.

^11^defined as the occurrence of one or more of the following complications: gestational diabetes, pre-eclampsia, fetal growth restriction, proteinuria, thrombopenia, threatened preterm labor, premature rupture of membranes (PROM), deep vein thrombosis and cholestasis of pregnancy.

^12^Small for gestational was defined by a birthweight < to the 10^th^ percentile according to the WHO fetal growth charts.

Additional table 4: Association between the three vulnerability indexes prenatal care use and poor pregnancy outcomes.

|  | **Inadequate PCU^1^** | |
| --- | --- | --- |
|  | **OR** | **aOR** |
| Administrative vulnerability index | 2.67 [2.38 – 3.01]*** | 2.53 [2.23 – 2.87]*** |
| Psychologic vulnerability index | 0.57 [0.47 – 0.70]*** | 0.62 [0.52 – 0.74]*** |
| Dependency vulnerability index | 1.18 [0.96 – 1.45] | 1.23 [1.02 – 1.46]* |
|  | **Premature birth (<37 weeks)** | |
|  | **OR** | **aOR** |
| Administrative vulnerability index | 1.49 [1.30 – 1.71]*** | 1.39 [1.19 – 1.61]*** |
| Psychologic vulnerability index | 3.08 [2.55 – 3.72]*** | 2.41 [1.98 – 2.95]*** |
| Dependency vulnerability index | 1.14 [0.91 – 1.43] | 1.34 [1.09 – 1.65]** |
|  | **Small for gestational age^2^** | |
|  | **OR** | **aOR** |
| Administrative vulnerability index | 1.29 [1.15 – 1.44]*** | 1.27 [1.13 – 1.43]*** |
| Psychologic vulnerability index | 1.32 [1.13 – 1.53]*** | 1.23 [1.05 – 1.44]** |
| Dependency vulnerability index | 1.00 [0.84 – 1.19] | 0.99 [0.84 – 1.17] |
|  | **Stillbirth** | |
|  | **OR** | **aOR** |
| Administrative vulnerability index | 1.52 [0.99 – 2.26] | 1.04 [0.62 – 1.65] |
| Psychologic vulnerability index | 4.85 [2.95 – 7.76]*** | 5.29 [2.88 – 9.78]*** |
| Dependency vulnerability index | 0.68 [0.31 – 1.43] | 1.00 [0.52 – 1.81] |
|  | **Late miscarriage** | |
|  | **OR** | **aOR** |
| Administrative vulnerability index | 0.78 [0.42 – 1.34] | 0.60 [0.29 – 1.11] |
| Psychologic vulnerability index | 3.58 [2.01 – 6.10]*** | 5.89 [2.84 – 12.33]*** |
| Dependency vulnerability index | 0.83 [0.36 – 1.80] | 0.87 [0.37 – 1.89] |
|  | **Medical abortion** | |
|  | **OR** | **aOR** |
| Administrative vulnerability index | 1.78 [1.04 – 2.87]* | 1.77 [0.94 – 3.09] |
| Psychologic vulnerability index | 5.41 [2.88 – 9.70]*** | 5.21 [2.48 – 11.11]*** |
| Dependency vulnerability index | 1.27 [0.49 – 2.94] | 1.62 [0.78 – 3.18] |

^1^pregnancy follow-up began after 12 weeks of gestation, or if it included less than 50% of the number of prenatal visits expected according to duration of pregnancy, or if the first-trimester ultrasound examination or both the second- and third- trimester examinations were missing.

^2^Small for gestational was defined by a birthweight < to the 10^th^ percentile according to the WHO fetal growth charts.

OR: Odd Ratio, OR were calculated for a one point increase in each vulnerability indexes.

aOR: adjusted Odd Ratio, adjustment on maternal age, maternal origin, parity, maternal body mass index, high medical risk level before pregnancy, high obstetrical risk level before pregnancy and the vulnerability indexes.

*p<0.05, **p<0.01,***p<0.001.
